# Probiotic *Bacillus subtilis* LF11 Protects Intestinal Epithelium Against *Salmonella* Infection

**DOI:** 10.3389/fcimb.2022.837886

**Published:** 2022-02-16

**Authors:** Rongling Zhang, Zhengguang Li, Xinyi Gu, Jiancun Zhao, Tingting Guo, Jian Kong

**Affiliations:** ^1^ State Key Laboratory of Microbial Technology, Shandong University, Qingdao, China; ^2^ Research and Development Center, Jinan Scenk Sanfeng Bioengineering Co., Ltd, Jinan, China

**Keywords:** *Bacillus subtilis*, intestinal barrier, *Salmonella* infection, exclusion activity, inflammatory cytokines, tight junction

## Abstract

Enteric diseases caused by *Salmonella* are prevalent in poultry farming. With the forbiddance of antibiotics in feedstuff industry, *Bacillus subtilis* (*B. subtilis*) preparation as antibiotic alternatives against *Salmonella* infection has gained increasing attention recently. However, the protection modes of *B. subtilis* against *Salmonella* infection in broilers are strain-specific. In this study, probiotic *B. subtilis* LF11 significantly reduced diarrhea and mortality of broilers caused by *Salmonella braenderup* (*S. braenderup*) in spite of no inhibition effect on it *in vitro*. Here, the intestinal epithelial cells NCM460 were incubated to explore the protection of *B. subtilis* LF11 on intestinal epithelium against *Salmonella*. The results revealed that *B. subtilis* LF11 showed obvious exclusion activity with the decrease of adhesion and invasion of *S. braenderup* to NCM460 cells, accordingly with the increase of NCM460 cell survival compared with *S. braenderup* challenge alone. Meanwhile, RT-PCR and Western blot proved that the gene transcription and expression levels of four tight junction proteins in NCM 460 cells were upregulated, which was further confirmed by immunofluorescence observation. Besides, *B. subtilis* LF11 downregulated the gene transcription levels of the proinflammatory cytokines IL-6, IL-8, and TNF-α induced by *S. braenderup* H9812. ELISA analysis also verified that *B. subtilis* LF11 reduced the IL-8 production significantly. In general, *B. subtilis* LF11 has the ability to protect the intestinal epithelium against *Salmonella* infection by reducing the *Salmonella* adhesion and invasion, enhancing the intestinal barrier and attenuating the enterocyte inflammatory responses, and has the potential as probiotics to prevent enteric diseases in broilers.

## Introduction

Intestinal barrier as the first line of defense between the host and the luminal environment plays a key role on the absorption of nutrients and adequate containment of microorganisms and molecules, which consists of mucus layer, intestinal epithelium, and intraepithelial immune cells (Wlodarska and Finlay, 2010; Sanchez de Medina et al., 2014; Awad et al., 2017). The damage of the intestinal barrier results in the permeability increasement and/or bacterial translocation, which in turn induces various enteric diseases (Natividad and Verdu, 2013; Sanchez de Medina et al., 2014). In poultry farming, primarily of chickens, pullorum disease and fowl typhoid caused by *Salmonella gallinarum* and *Salmonella pullorum*, respectively, are the most common enteric diseases, resulting in severe economic losses (Crump et al., 2015; Arreguin-Nava et al., 2019; Lei et al., 2020; Chen et al., 2021). *Salmonella* infection involves normally the attachment to epithelial cells, the disruption of intestinal barrier, and internalization into lamina propria, and consequently leads to the inflammatory responses and even induces diarrhea and death in broilers (Bruno et al., 2009; Gut et al., 2018; Wang et al., 2018). Using of antibiotics as a traditional way to prevent and treat enteric diseases has led to the evolution of resistant strains of pathogenic bacteria (Nami et al., 2015; Lei et al., 2020). The forbiddance of antibiotic growth promoters facilitates the development of the functional feed additives as potential alternatives for antibiotics to avoid the incidence of bacterial resistance (Oh et al., 2017).

Probiotics is gaining increasing attention as an antibiotic alternative used in the poultry husbandry (Nami et al., 2015; de Melo Pereira et al., 2018). Among the various probiotic strains, *B. subtilis* is the major strain widely used as a functional feed additive (Elshaghabee et al., 2017; Du et al., 2019; Popov et al., 2021). This species possesses well-known industrial traits and physiological characteristics (Elshaghabee et al., 2017; Popov et al., 2021). The tolerance to harsh environments such as pH, bile salts, and heat endows *B. subtilis* high viability in the intestinal tracts, processing, and storage (Lee et al., 2017). Additionally, *B. subtilis* produces a variety of digestive enzymes, antimicrobial peptides and other biological active molecules, improving the growth performance of broilers and feed efficiency when supplemented in diets (Manhar et al., 2016; Ramlucken et al., 2019; Akhtar et al., 2021; Algburi et al., 2021; Popov et al., 2021). Moreover, *B. subtilis* is beneficial to the host health through modulation of microbiota composition and humoral immune responses (Khan and Chousalkar, 2020; Kawarizadeh et al., 2021). Recently, several clinical and animal trials have demonstrated the protection of *B. subtilis* against *Salmonella* and other enteropathogenic bacteria in broilers through the load reduction in intestinal tracts, improvement of the growth performance, and immune responses (Wu et al., 2018; Sokale et al., 2019; Abudabos et al., 2020). Nevertheless, the action modes of probiotic *B. subtilis* are in a strain-specific manner (Freedman et al., 2021), which makes it necessary to investigate the potential protection against *Salmonella* infection in poultry farming.

In this study, *B. subtilis* LF11 strain, isolated from corn silage, showed the obvious inhibition activity against Gram-positive pathogenic bacteria, not for *Salmonella*, whereas the administration of *B. subtilis* LF11 could decrease the diarrhea and mortality of broilers, suggesting the protective role on broilers against *Salmonella* infection. To investigate the protection mechanism of this strain, the cultured intestinal epithelial cells NCM460 were exposed to strains *B. subtilis* LF11, and followed by the challenge with *S. braenderup* H9812. The gene transcription and expression levels of the tight junction proteins and the proinflammatory factor were detected by RT-PCR and Western blot. The aim of this study is to reveal the protection of *B. subtilis* LF11 against *Salmonella* infection and to prove its potential as an antibiotic alternative to prevent the enteric diseases in broilers.

## Materials and Methods

The animal study was reviewed and approved by the Animal Ethics Committee of School of Life Sciences, Shandong University.

### Strains, Cells, and Culture Conditions

*B. subtilis* LF11 was isolated from corn silage previously (Li, 2019); *S. braenderup* H9812 and other indicator strains *Escherichia coli* (*E. coli*) ATCC 25922, *Listeria monocytogenes* (*L. monocytogenes*) ATCC 19114, *L. monocytogenes* ATCC 19115, *Staphylococcus aureus* (*S. aureus*) ATCC 27217, *Streptococcus agalactiae* (*S. agalactiae*) ATCC 13813, *Micrococcus luteus* (*M. luteus*) 2016, *Bacillus cereus* (*B. cereus*) SDMCC 050292, *Enterococcus faecalis* (*E. faecalis*) SDMCC 050338, *Streptococcus lutetiensis* (*S. lutetiensis*) SDMCC 050401, and *Streptococcus infantarius* (*S. infantarius*) SDMCC 050384 were stored in our laboratory and cultured according to methods described previously (Parveen Rani et al., 2016). *B. subtilis* LF11 was grown in Luria-Bertani (LB) broth (Oxord, Basingstoke, UK) at 37°C, shaking at 150 rpm overnight. Two percent of overnight cultures was inoculated in 5 ml of fresh LB broth and incubated for 12 h. The cells were collected by centrifugation at 6,000 rpm for 5 min at 4°C and the cell-free culture supernatants (CFCS) were collected by centrifugation at 10,000 × *g* for 20 min at 4°C and filtration through a 0.22 μm membrane filter (Millipore, USA). The enterocyte NCM460 cells were cultured in RPMI 1640 medium (SparkJade, Shandong, CHN) supplemented with 10% (v/v) fetal calf rerum and 1% penicillin/streptomycin (penicillin: 10,000 U/ml, streptomycin: 10,000 µg/ml, Gibco-Life Technologies, Carlsbad, CA, USA) at 37°C in a 5% CO_2_ atmosphere.

### Broilers Feeding Trial

A total of two hundred 1-day-old male Ross-308 broiler chicks free of *Salmonella* was purchased from a commercial hatchery (Shandong Dabao, Tai’an, CHN). The chicks were individually weighed and assigned to 5 groups with 4 replicates (10 birds per pen). The treatment groups were as follows: negative control group (NC) was given basal diet only; positive control group (PC) was given basal diet and orally administered with 0.1 ml of *S. braenderup* H9812 suspension (1×10^9^ CFU/ml) on day 7; *B. subtilis* control group (BC) was given only probiotic-supplemented basal diet containing *B. subtilis* LF11 to a final concentration of 1×10^9^ CFU/kg feed; antibiotic-treated group (AT) was orally challenged by 0.1 ml of *S. braenderup* H9812 suspension above on day 7, administered in basal diet supplemented with amoxicillin (Jinan Scenk, Jinan, CHN) at its highest recommended dose (0.24 g/kg) on the day when clinical signs first appeared and continued for 5 days (Gharaibeh et al., 2021). The *B. subtilis*-treated group (BT) was given probiotic-supplemented basal diet containing *B. subtilis* LF11 to the same concentration above and orally challenged on day 7 with 0.1 ml of *S. braenderup* H9812 suspension. To avoid cross-contamination, the uninfected chicks and infected chicks were reared in two separate rooms respectively equipped with battery cages (1.0 m× 0.7 m × 0.5 m, length × width × height) with stocking density at 0.07 m^2^ per chick. The room temperature was maintained at 32 ± 2°C during the first week and then gradually decreased by 3°C weekly until a final room temperature of 26°C. Antibiotic-free and coccidiostat-free corn-soybean meal-based mash diets were prepared to meet the requirements for starter and grower periods. The composition of the basal diet and nutrient levels are prepared as described by Wu et al. (2018). All birds were allowed *ad libitum* access to feed and water throughout the study. In addition, the chickens were vaccinated against Newcastle disease virus (NDV) and infectious bronchitis virus (IBV) according to the antiepidemic recommendations (Abudabos et al., 2020).

Broilers with diarrhea or death were counted and the feces was recorded every day during the experiment. Mortality rate per treatment was calculated for the entire trial period. Weights of all died birds throughout the trial period were recorded. Body weight (BW) and feed intake (FI) were recorded weekly (days 0, 7, 14, 21, and 28) as an average per pen. Feed conversion ratio (FCR) adjusted for mortalities was calculated for each pen as FI (g)/BW gain (g) over a specified period of time.

### Determination of Antimicrobial Spectrum

Antimicrobial spectrum was determined by agar well-diffusion method (Reuben et al., 2019). Briefly, 100 μl of indicator strain (approximately 10^8^ CFU/ml) was mixed with 10 ml of LB agar, or tryptic soytone broth (TSB) agar, or brain heart infusion (BHI) agar and poured into a Petri dish covered with 10 ml of water agar beforehand. After solidification, the wells were prepared and filled with 50 μl of the CFCS of *B. subtilis* LF11. The plates were cultured at 37°C for 24 h, and the inhibition zone diameters were measured.

### Determination of the Counts of *Salmonella* Adhering to or Invading NCM460 Cells

The counts of *S. braenderup* H9812 adhering to and invading NCM460 cells were enumerated as described previously (Tsai et al., 2005; Tuo et al., 2013) with few modifications. Briefly, *B. subtilis* LF11 and *S. braenderup* H9812 cells were suspended with RPMI 1640 medium without antibiotics, and NCM460 cells grown in a 12-well plate (1×10^5^ cells/well) were washed twice with phosphate buffer saline (PBS, pH 7.2). In an exclusion experiment, NCM460 monolayers were incubated with *B. subtilis* LF11 at a multiplicity of infection (MOI) of 100 and then challenged by *S. braenderup* H9812 at the same MOI. In a competition experiment, NCM460 monolayers were incubated simultaneously with *B. subtilis* LF11 and *S. braenderup* H9812 at the MOI of 100. All the incubations were carried out for 2 h at 37°C in 5% CO_2_. To determine the counts of *S. braenderup* H9812 adhering to NCM460 cells, the monolayers were washed 4 times with sterilized PBS (pH 7.2), and then 100 μl of 0.5% (v/v) Triton X-100 solution was added to the wells to detach the bacteria. The counts of *S. braenderup* H9812 were determined by spreading serial dilutions on bismuth sulfite agar (BSA) plates (Hope Bio-tech, Qingdao, CHN) (Manhar et al., 2016). For the invasion count determination, the monolayer cells were incubated for 2 h with 100 μl of gentamicin solution (200 μg/ml) to kill the bacteria outside of the cells. Then, NCM460 cells were lysed by adding sterilized water. The counts of *S. braenderup* H9812 invading cells were detected using the same method as described above. The exposure of NCM460 cells to *S. braenderup* H9812 alone was set as a control. Data were expressed as mean ± SD.

### Evaluation of NCM460 Cell Survivability

The survival of NCM460 cells was evaluated after *B. subtilis* LF11 and *S. braenderup* H9812 incubation in exclusion and competition experiments by the cell counting kit-8 (cck-8) assay according to the instructions (US Everbright^®^ Inc, Suzhou, CHN). NCM460 monolayers were grown in a 96-well plate and treated for 6 h at the MOI of 100. Then 100 μl of gentamicin solution (200 μg/ml) was added to the wells and incubated for 2 h at 37°C to kill the bacteria. Subsequently, a total of 10 μl (5 mg/ml) of cck-8 and 100 μl of RPMI 1640 medium were added and incubated for 2 h. Optical density (OD) was detected at 450 nm. The cells treated or not treated by *S. braenderup* H9812 alone were used as controls. Six parallels were set for each treatment. Data were expressed as mean ± SD.

### Analysis of the Gene Transcription Levels of Tight Junction Proteins and Proinflammatory Cytokines

Primers used in this study are listed in **Table 1**. In exclusion and competition experiments at the MOI of 100, NCM460 cells were washed twice with pre-cooling PBS (pH 7.2) and collected by centrifugation at 1,000 rpm for 5 min at 4°C after dissociation by 0.25% trypsin-Ethylene Diamine Tetraacetic Acid (EDTA) solution. Total RNA was extracted with SPARKeasy Cell RNA Kit (SparkJade, Jinan, CHN) and the cDNA was synthesized using the PrimeScript RT reagent kit (Takara, Dalian, CHN). The gene transcription levels of tight junction proteins including occludin (OCLN), claudin-1 (CLDN-1), junctional adhesion molecule-1 (JAM-1), and the zonulae occludens-1 (ZO-1) and including proinflammatory cytokines interleukin-6 (IL-6), interleukin-8 (IL-8), and tumor necrosis factor-α (TNF-α) were analyzed by RT-PCR, which was carried out with an iCycler kit (ABclonal, Wuhan, CHN). PCR amplification was carried out as follows: initial denaturation at 95°C for 1 min followed by 35 cycles at 95°C for 5 s, 55°C for 30 s, and 72°C for 30 s (Sun et al., 2012). The *β-actin* was used as the housekeeping gene, and the data of relative gene transcription were analyzed using the 2^−ΔΔCt^ method as previously described (Livak and Schmittgen, 2001). The untreated NCM460 cells were set as control.

**Table 1:** Primer sequences used for RT-PCR.

Gene	Primer sequence (5’ to 3’)	Source or Reference
*β-actin*-Forward	TACATCACTATTGGCAACGAGC	(Wang et al., 2019)
*β-actin*-Reverse	GTCGGATGTCAACGTCACACTT	(Wang et al., 2019)
*IL-6*-Forward	CACTGGTCTTTTGGAGTTTGAG	This study
*IL-6*-Reverse	GGACTTTTGTACTCATCTGCAC	This study
*IL-8*-Forward	ATACTCCAAACCTTTCCACCC	This study
*IL-8*-Reverse	AACTTCTCCACAACCCTCTGC	This study
*TNF-α*-Forward	CGTGGAGCTGGCCGAGGAG	This study
*TNF-α*-Reverse	GCAGGCAGAAGAGCGTGGTG	This study
*CLDN-1*-Forward	GTGCCTTGATGGTGGTTG	(Liu et al., 2011)
*CLDN-1*-Reverse	TGTTGGGTAAGAGGTTGT	(Liu et al., 2011)
*OCLN*-Forward	GCAGCTACTGGACTCTACG	(Liu et al., 2011)
*OCLN*-Reverse	ATGGGACTGTCAACTCTTTC	(Liu et al., 2011)
*ZO-1*-Forward	AAGAGTGAACCACGAGAC	(Liu et al., 2011)
*ZO-1*-Reverse	TCCGTGCTATACATTGAG	(Liu et al., 2011)
*JAM-1*-Forward	GATGTGCCTGTGGTGCTG	(Liu et al., 2011)
*JAM-1*-Reverse	GCTCTGCCTTGAGATAAGAA	(Liu et al., 2011)

### Western Blot and Immunofluorescence Assay

The expression levels of tight junction proteins CLDN-1, OCLN, JAM-1, and ZO-1 in NCM460 cells were detected by Western blot according to Liu et al. (2010). NCM460 monolayers were harvested as described above and the total proteins were extracted according to the manufacturer instructions (Beyotime, Shanghai, CHN). The proteins were separated by 12% sodium dodecyl sulfate (SDS) polyacrylamide gel electrophoresis and transferred onto polyvinylidene difluoride membrane (Millipore, Bedford, MA, USA) at 200 mA in transfer buffer (192 mM glycine and 25 mM Tris). The membrane was blocked for 1 h with tris buffered saline tween (TBST) buffer containing 5% skim milk. Strip membranes at corresponding protein size were cut off and probed overnight at 4°C with rabbit anti-CLDN-1 (1:1500, ABclonal, CHN), rabbit anti-OCLN (1:1500, ABclonal, CHN), rabbit anti-JAM-1 (1:1500, ABclonal, CHN), rabbit anti-ZO-1 (1:1500, ABclonal, CHN), and rabbit anti-β-actin (1:2000, ABclonal, CHN), correspondingly. After washing with TBST buffer, the membranes were incubated for 1 h with the horseradish peroxidase-conjugated secondary antibody (1:5000, ABclonal, CHN). The Amersham Imager 680 (GE, USA) was used to capture images and analyze the gray values of CLDN-1, OCLN, JAM-1, and ZO-1 protein as normalized to β-actin.

The expression and distribution of tight junction proteins were observed using an immunofluorescence microscope. Briefly, NCM460 monolayers were fixed with 2% (v/v) acetone/methanol on ice after treatments as described above and permeated by 0.2% Triton X-100. The cells were probed overnight at 4°C with primary antibodies above and then incubated with the fluorescein isothiocyanate-conjugated secondary antibody for 1 h. Nuclei were stained with DAPI (Beyotime, Shanghai, China). The cells were observed by fluorescence microscope in the anti-attenuation reagent. Quantitative analysis of fluorescence was carried out using ImageJ (Jensen, 2013).

### IL-8 ELISA Assay

After incubation of NCM460 cells in exclusion and competition experiments, the suspension was collected by centrifugation at 12,000 rpm for 5 min at 4°C, and the concentration was detected as the instruction of human IL-8 ELISA kit (ABclonal Tech^®^, CHN).

### Statistical Analysis

The experiments were performed in triplicate. Statistical analysis was performed using unpaired two-tailed Student’s *t*-tests. The significance between treatments was determined by an analysis of variance with the general linear model. *p*-values were considered statistically significant. *p < 0.05, **p < 0.01. Differences between treatments for mortalities were calculated with a chi square.

## Results

### *B. subtilis* LF11 Reduced the Diarrhea and Death of Broilers Challenged by *Salmonella*

Challenge trials were carried out to validate whether *B. subtilis* LF11 protects the broilers from *Salmonella* or not. The results are listed in **Table 2**. A few chickens appeared to have slight diarrhea but none died in every group before 7 days old. Diarrhea symptoms disappeared and no death chickens appeared in NC and BC groups from then on. Diarrhea occurred in all groups challenged by *S. braenderup* H9812 at about 10 days old, and the PC group was most serious, whose excrements were chalky white. Diarrhea rate and mortality in PC group increased to 67.5 % and 42.5 % respectively at the end of the trial. Though only 5% of diarrhea and no death appeared in the AT group that were treated with amoxicillin between 11 and 15 days old because of the therapeutical effect of antibiotics, diarrhea rate and mortality were rising again at a withdrawal time of up to 20% and 15%. Correspondingly, the ascensional ranges of diarrhea and death in the BT group were higher than that in the AT group but lower than that in the PC group obviously. No broilers died and diarrhea declined in the BT group after 21 days old, of which solid content increased and chalky white diarrhea reduced. Additionally, numerical improvements in FCR (1.297 g/g) and BW gain (1.157 kg) of BC group at 28 days old were not statistically significant compared with the negative control group. The differences in FCR (1.358 g/g, and 1.404 g/g, respectively) and BW gain (1.141 and 1.105 kg, respectively) in AT and BT groups were statistically significant compared with the positive control group.

**Table 2:** *B. subtilis* LF11 reduced the diarrhea and death of broilers challenged by *Salmonella*.

Treatment		Day-old					FCR (g: g)
		1	7	14	21	28	
NC	Diarrhea rate (%)	0	2.5	0	0	0	1.333
	Mortality (%)	0	0	0	0	0
	BW (g)	52.4±	139.7	360.1	696.1	1,118.3
PC	Diarrhea rate(%)	0	2.5	45	55	67.5	1.506
	Mortality (%)	0	0	25	30	42.5
	BW (g)	52.6	140.9	321.9	600.2	981
BC	Diarrhea rate (%)	0	5	0	0	0	1.297**
	Mortality (%)	0	0	0	0	0
	BW (g)	52.5	143.8	376	717.1	1,156.6*
AT	Diarrhea rate (%)	0	0	5	12.5	20	1.358**
	Mortality (%)	0	0	0	7.5	15
	BW (g)	52.4	138.1	346.4	676.4	1,140.8*
BT	Diarrhea rate (%)	0	2.5	15	32.5	25	1.404**
	Mortality (%)	0	0	15	17.5	17.5
	BW (g)	52.4	145	357.3	688.9	1,105.3*

NC, basal diet only; PC, basal diet + S. braenderup H9812; BC, basal diet + B. subtilis LF11; AT, basal diet + S. braenderup H9812 + amoxicillin; BT, basal diet + B. subtilis LF11 + S. braenderup H9812; BW, body weight; FCR, feed conversion ratio. *p < 0.05, **p < 0.01 vs. PC group.

### The Antimicrobial Spectrum of *B. subtilis* LF11

The antimicrobial activity was generally considered as a vital assessment criterion for probiotics screening. As shown in **Table 3**, most of the Gram-positive bacteria tested were inhibited by the CFCS of *B. subtilis* LF11, especially the pathogens *L. monocytogenes* ATCC 19114, *L. monocytogenes* ATCC 19115, and *S. agalactiae* ATCC 13813, but not for Gram-negative pathogenic bacteria *Escherichia coli* (*E. coli*) ATCC 25922 and *S. braenderup* H9812.

**Table 3:** Inhibitory spectrum of *B. subtilis* LF11.

Indicator strains	Antimicrobial ability^a^
Gram-positive bacteria	
*M. luteus* 2016	+++
*L. monocytogenes* ATCC 19114	+++
*L. monocytogenes* ATCC 19115	+++
*S. aureus* ATCC 27217	–
*B. cereus* SDMCC 050292	+
*E. faecalis* SDMCC050338	–
*S. agalactiae* ATCC 13813	+++
*S. lutetiensis* SDMCC050401	++
*S. infantarius* SDMCC050384	++
Gram-negative bacteria	
*E. coli* DH5α	–
*E. coli* ATCC 25922	–
*S. braenderup* H9812	–

a Symbols show the antimicrobial ability of B. subtilis LF11: –, no inhibition; +, weak (Diameter of bacteriostatic zone <14 mm); ++, good (Diameter of bacteriostatic zone 15–19 mm); +++, strong (Diameter of bacteriostatic zone >20 mm).

### *B. subtilis* LF11 Inhibited *S. braenderup* H9812 Adhesion and Invasion to NCM460 Cells

**Figure 1:**
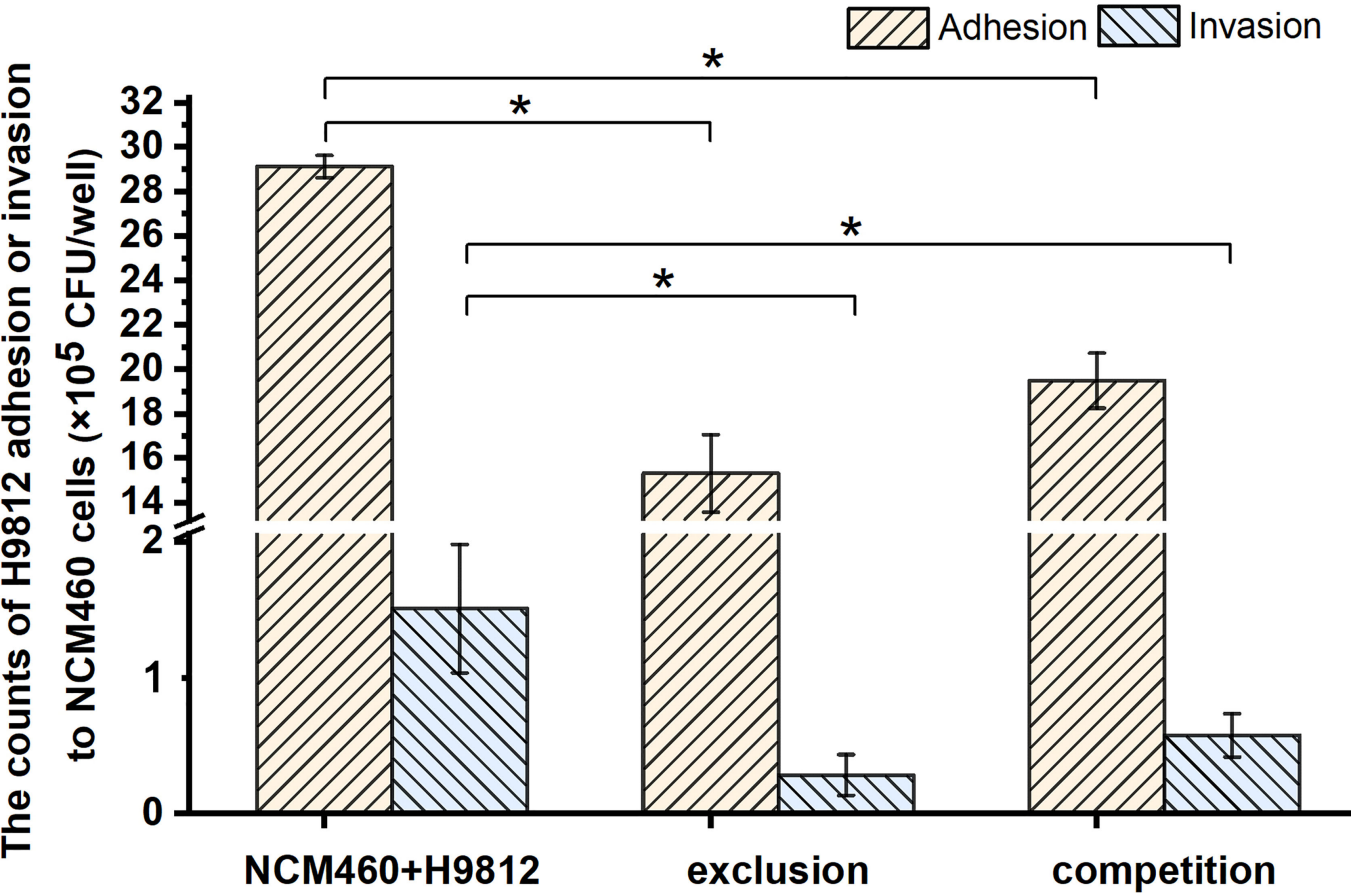
The bacterial counts of *S. braenderup* H9812 adhering to and invading NCM460 cells. All data were given as means ± SD for *n* = 3. *p < 0.05 vs. NCM460 + H9812.

The adhesion and invasion of *Salmonella* to IECs are the important steps to its pathogenicity and translocation. To investigate the protective role of *B. subtilis* LF11 against *Salmonella* infection in broilers, the intestinal epithelial cell NCM460 was adopted as a mode to test the ability of *B. subtilis* LF11 to inhibit the adhesion and invasion of the pathogen *S. braenderup* H9812 to NCM460 cells *in vitro*. The results showed that the adhesion and invasion of *S. braenderup* H9812 to NCM460 cells were up to (2.91 ± 0.05) ×10^6^ CFU/well and (1.51 ± 0.47) ×10^5^ CFU/well when NCM460 cells were exposed to *S. braenderup* H9812 alone (**Figure 1**). In the exclusion experiment, the strain LF11 exhibited the obvious exclusive ability, preventing 47.4% and 81.1% of *S. braenderup* H9812 from the adhesion and invasion to NCM460 cells (*p* < 0.05). In competition experiment, the strain LF11 also prevented the adhesion and invasion of the pathogenic strain H9812 to NCM460 cells by 33.1% and 61.7% (*p* < 0.05). Both experimental data demonstrated the inhibition effects of *B. subtilis* LF11 on *S. braenderup* H9812 adhesion and invasion to NCM460 cells, and the exclusion activity was stronger than competition activity.

### *B. subtilis* LF11 Increased Survivability of NCM460 Cells Challenged by *S. braenderup* H9812

**Figure 2:**
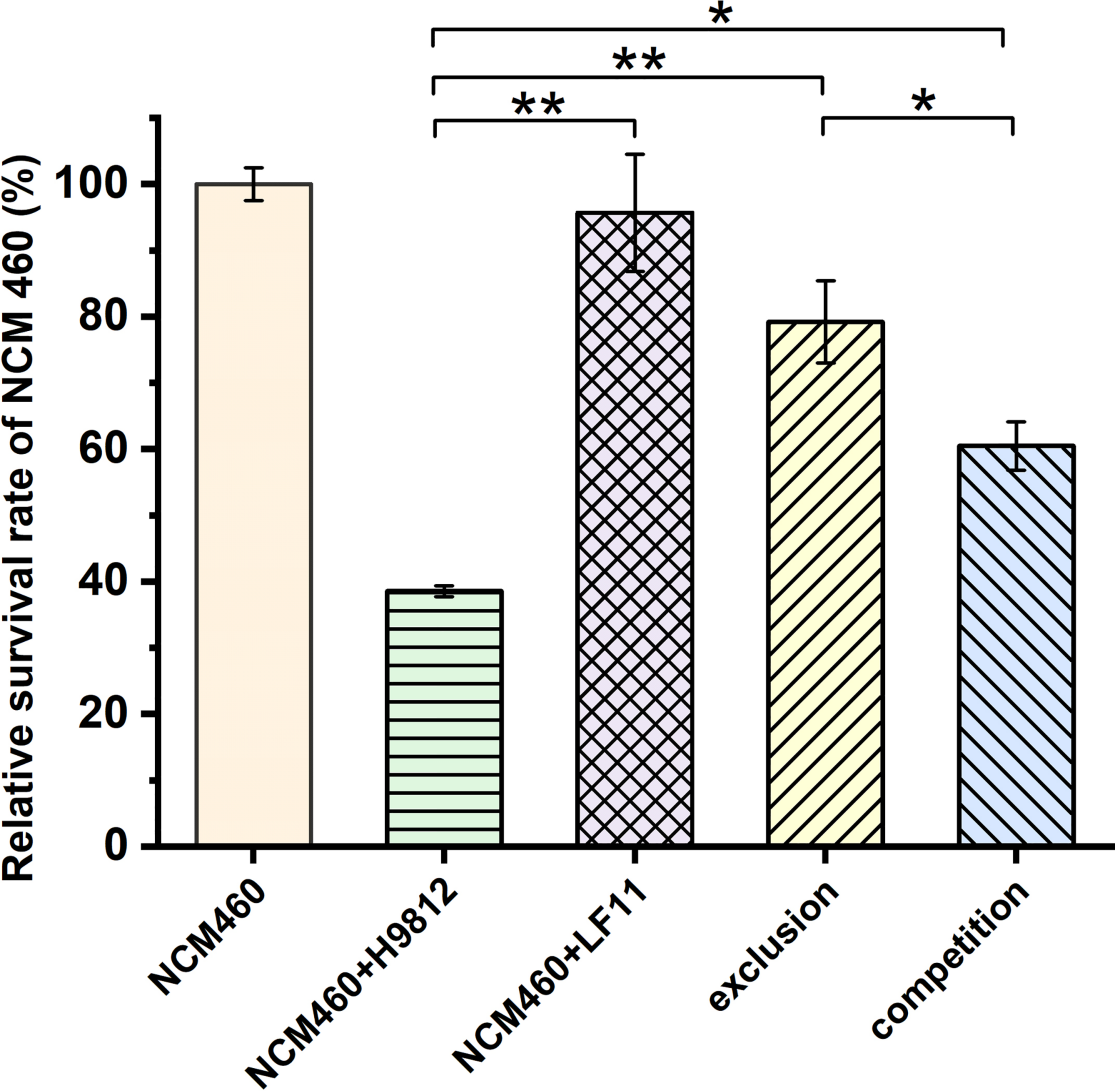
The relative survivability of NCM460 cells with various treatments. The survival of the normal NCM460 cells is defined as 100%. All data are given as means ± SD for *n* = 3. Statistical analysis is performed with Student’s *t*-test. *p < 0.05, **p < 0.01.

To confirm the inhibition of *B. subtilis* LF11 against *S. braenderup* H9812 invasion to NCM460 cells, the survivability of NCM460 cells was measured in exclusion and competition experiments. As shown in **Figure 2**, no significant difference was observed between the survivability of NCM460 cells alone and those exposed to *B. subtilis* LF11, indicating that *B. subtilis* LF11 did not influence the survival of the IECs. However, when NCM460 cells were exposed to *S. braenderup* H9812 alone, the survival of NCM460 cells was decreased to (38.54 ± 0.85)%, indicating that *S. braenderup* H9812 was fatal to NCM460 cells. In the exclusion experiment, the survivability of NCM460 cells was improved to (79.2 ± 6.21)%, higher than (60.5 ± 3.68)% in the competition experiment, suggesting that the pre-exposure to *B. subtilis* LF11 could reduce the damage of NCM460 cells caused by *S. braenderup* H9812 (*p* < 0.05).

### *B. subtilis* LF11 Enhanced the Tight Junction of *Salmonella*-Infected NCM460 Cells

**Figure 3:**
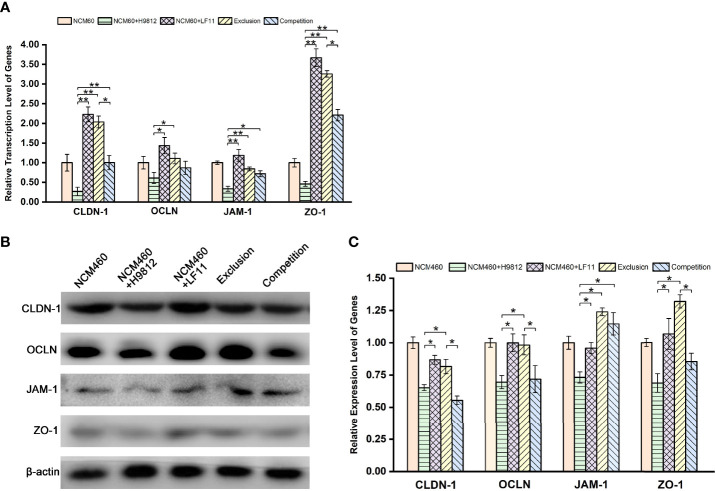
*B. subtilis* LF11 enhances the gene transcription and expression of tight junction proteins. **(A)** Relative transcription levels of *CLDN-1*, *OCLN*, *JAM-1*, and *ZO-1* genes. **(B)** Expression levels of tight junction proteins in NCM460 cells incubated with *B. subtilis* LF11 and *S. braenderup* H9812 with various treatments by Western blot. Each line represents different treatments. **(C)** Semiquantitative analysis of the expression of tight junction proteins in corresponding bands. The β-actin was used as the housekeeping gene, the data of relative gene transcription and expression levels were analyzed using the 2^−ΔΔCt^ method, and the gene transcription or expression levels of tight junction proteins in control NCM460 cells are defined as 1.0. All data are given as means ± SD for *n* = 3. *p < 0.05, **p < 0.01.

Intestinal barrier dysfunction exerts a pivotal physiological role in the development of enteric diseases. To further evaluate the molecular mechanism underlying the *B. subtilis* LF11 protection of the intestinal barrier, the gene transcription levels of tight junction proteins CLDN-1, OCLN, JAM-1, and ZO-1 in NCM460 cells were detected by RT-PCR. *S. braenderup* H9812 downregulated respectively the gene transcription levels of those tight junction proteins to 0.27 ± 0.11, 0.61 ± 0.14, 0.34 ± 0.06, and 0.46 ± 0.06 compared with that of NCM460 cells alone. However, *B. subtilis* LF11 significantly upregulated the transcription levels of tight junction genes in both exclusion and competition experiments, as well as exposed to *B. subtilis* LF11 alone. Particularly, *B. subtilis* LF11 upregulated apparently the gene transcription levels of *CLDN-1*, *JAM-1*, and *ZO-1* (*p* < 0.01), and correspondingly *OCLN* (*p* < 0.05). The effect of *B. subtilis* LF11 to CLDN-1 and ZO-1 in exclusion experiment was obviously higher than those in the competition experiment (**Figure 3A**).

The expression levels of the tight junction proteins in NCM460 cells were further confirmed by Western blot and gray value analysis. As shown in **Figures 3B, C**, *B. subtilis* LF11 did not influence the expression of the tight junction proteins apparently, while *S. braenderup* H9812 downregulated the expression of those proteins obviously (*p* < 0.05). *B. subtilis* LF11 restored the expression levels of CLDN-1, OCLN, JAM-1, and ZO-1 protein to 0.82 ± 0.06, 0.98 ± 0.08, 1.23 ± 0.03, and 1.32 ± 0.05, respectively, in exclusion experiments in comparison with 0.65 ± 0.02, 0.69 ± 0.05, 0.73 ± 0.04, and 0.68 ± 0.07 in the *S. braenderup*-treated group (*p* < 0.05). However, the changes of the expression levels of tight junction proteins in competition experiments did not show statistical significance (*p* > 0.05) except for JAM-1 (**Figures 3B, C**). The expression levels of JAM-1 protein in exclusion and competition experiments did not represent statistical difference.

**Figure 4:**
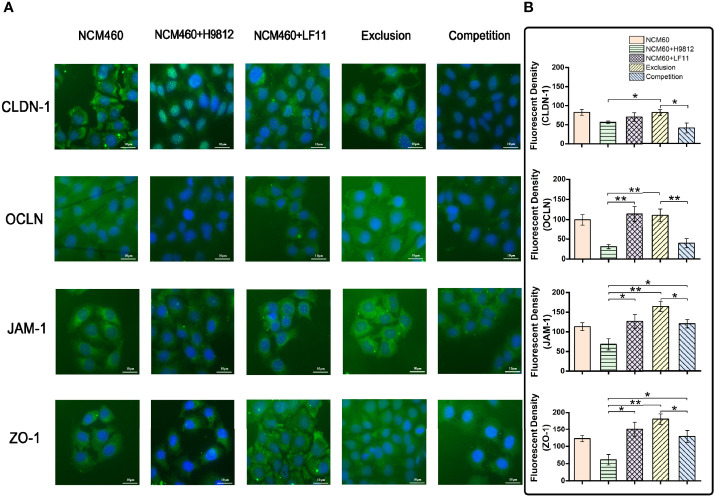
The immunofluorescence analysis of tight junction proteins in NCM460 cells. **(A)** Expression levels and distribution of these tight junction proteins in NCM460 cells with various treatments are visualized by immunostaining fluorescein isothiocyanate (FITC, green). Nuclei are counterstained using 40,6-Diamidino-2-phenylindole dihydrochloride (DAPI, blue). **(B)** Quantitative analysis of fluorescence density was carried out using ImageJ. Statistical analysis is performed with Student’s *t*-test. Scale bar = 10 μm. *p < 0.05, **p < 0.01.

The expression and distribution of the tight junction proteins in NCM460 cells with different treatments were observed directly by the immunofluorescence microscope. As shown in **Figures 4A, B**, the homogeneous distribution and the fluorescence intensity of the four tight junction proteins in *B. subtilis* LF11-treated and exclusion experiments were almost the same levels as the normal NCM460 cells, which appeared stronger than that treated with *S. braenderup* H9812 alone. However, *B. subtilis* LF11 did not recover the expression and distribution of CLDN-1 and OCLN proteins to normal state in competition experiments but JAM-1 and ZO-1 did. These results were consistent with the expression levels of tight junction proteins in Western blot analysis.

### *B. subtilis* LF11 Attenuated the Expression Levels of the Proinflammatory Genes Induced by *S. braenderup* H9812

**Figure 5:**
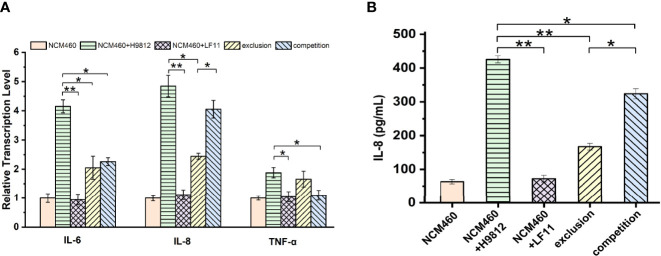
*B. subtilis* LF11 attenuates the expression of the proinflammatory cytokines. **(A)** Relative gene transcription levels of three proinflammatory cytokines in NCM460 cells in various treatments. The β-actin was used as the housekeeping gene, the data of relative gene transcription and expression levels were analyzed using the 2^-ΔΔCt^ method, and the gene transcription levels of three proinflammatory cytokines in control NCM460 cells are defined as 1.0. **(B)** IL-8 production in NCM460 cells induced by various treatments with *B. subtilis* LF11, *S. braenderup* H9812 and their combinations. Error bars represented standard errors from three replicate experiments. *p < 0.05, **p < 0.01.

To investigate the effects of *B. subtilis* LF11 on the inflammatory process of NCM460 cells induced by *S. braenderup* H9812, the gene transcription levels of three key proinflammatory cytokines IL-6, IL-8, and TNF-α involved in the proinflammatory responses were analyzed. As shown in **Figure 5A**, *S. braenderup* H9812 upregulated the gene transcription levels of *IL-6*, *IL-8*, and *TNF-α* in NCM460 cells by 4.2, 4.9, and 1.9-folds, respectively (*p* < 0.05), while *B. subtilis* LF11 did not. *B. subtilis* LF11 downregulated the gene transcription levels of *IL-6*, *IL-8*, and *TNF-α* in NCM460 cells by 50.7%, 49.7%, and 11.7% in exclusion experiments as well as by 45.6%, 16.3%, and 46.8% in competition experiments. Additionally, the transcription levels of *IL-6* gene were partially alleviated in both exclusion and competition experiments (*p* < 0.05), and the alleviation of the transcription level of *IL-8* gene in exclusion experiments was stronger than that in competition experiments, which was contrary to *TNF-α*.

IL-8 production was detected by ELISA to further clarify the anti-inflammatory effects of *B. subtilis* LF11. The IL-8 production in NCM460 cells exposed to *B. subtilis* LF11 was the same as in NCM460 cells alone, whereas *S. braenderup* H9812 stimulated the IL-8 secretion by approximate 7-fold. When NCM460 cells were preincubated with *B. subtilis* LF11 and then were infected by *S. braenderup* H9812, IL-8 production significantly decreased by 60.7% (*p* < 0.01), which reduced by 23.9% in the case of the co-incubation with *B. subtilis* LF11 and *S. braenderup* H9812 (**Figure 5B**). These results suggested that *B. subtilis* LF11 attenuated significantly the release of proinflammatory cytokines induced by *S. braenderup* H9812, and the exclusion activity of *B. subtilis* LF11 was stronger than the competition activity.

## Discussion

*Salmonella* infection is one of the most serious problems that threaten poultry health, particularly for the newborn chickens (Ferrari et al., 2019). With the forbiddance of antibiotics worldwide, probiotics as the ideal antimicrobial agents is demonstrated to confer the beneficial effects on improving the growth performance, reducing morbidity, and enhancing immunity in poultry husbandry instead of the antibiotic utilization as growth promotion agents (Cheng et al., 2014; EMA/EFSA, 2017). Based on the selection criteria “Guidelines for Evaluation of Probiotics in Food (FAO/WHO)” (de Melo Pereira et al., 2018), strain *B. subtilis* LF11 with the promising probiotic traits was isolated from corn silage. When *B. subtilis* LF11 was supplemented in broiler diets, the diarrhea and mortality rates caused by *Salmonella* were obviously reduced in broiler farming, demonstrating the protection of *B. subtilis* LF11 against *Salmonella* infection. Meanwhile, the improvement of feed intake and feces shape suggested that the integrity and barrier of the intestinal epithelium were repaired or not impaired (**Table 2**). However, this probiotic strain LF11 did not exhibit the inhibition activity on the pathogen *S. braenderup* H9812 and *E. coli* ATCC 25922 *in vitro* (**Table 3**). In this case, we hypothesized that the protective role of *B. subtilis* LF11 against *S. braenderup* H9812 infection might target the intestinal epithelium barrier rather than inhibiting the pathogen bacterial growth in gut of tested broilers. Therefore, to clarify the molecular mechanism, the effects of *B. subtilis* LF11 on *S. braenderup* H9812 adhesion and invasion as well as the IECs survivability were detected through the exclusion and competition experiments. Expectedly, *B. subtilis* LF11 was able to inhibit the adhesion and invasion of *S. braenderup* H9812 to IECs NCM460, facilitate the expression of the tight junction proteins of NCM460 cells, and attenuate the inflammatory responses of IECs, revealing the protection role of *B. subtilis* LF11 on broilers against *Salmonella* infection *via* repairing or improving the intestinal barrier and suppressing the intestinal inflammation.

In general, antimicrobial activity is the main mode of action of probiotics to prevent bacterial diseases (Compaore et al., 2013; Nami et al., 2015; Du et al., 2019; Algburi et al., 2021; Zhou et al., 2021). Because no inhibitory activity on the growth of the *S. braenderup* H9812 was detected *in vitro*, prevention of *B. subtilis* LF11 from *S. braenderup* H9812 infection might be caused by other possible mechanisms including intestinal barrier (Rao and Samak, 2013), coaggregation (Collado et al., 2007), and signal interference (Piewngam et al., 2018). As shown in **Figure 1**, the count decline of *S. braenderup* H9812 adhering to and invading NCM460 cells indicated that *B. subtilis* LF11 could reduce the *Salmonella* load in intestinal tracts of animals, which was consistent with the reports of animal testing (Knap et al., 2011; Kridtayopas et al., 2019; Nishiyama et al., 2021). Furthermore, the counts of *S. braenderup* H9812 adhering to and invading NCM460 cells were enormously dependent on the orders of NCM460 cells exposed to *B. subtilis* LF11, which suggested that *B. subtilis* LF11 played the roles under certain conditions. In the exclusion experiment, the adhesion counts of *S. braenderup* H9812 reduced by 47.4% while invasion counts reduced by 81.1%, suggesting that *B. subtilis* LF11 reduced the *Salmonella* invasion by enhancing the defense of NCM460 cells in addition to attenuating the adhesion. The same phenomenon of the invasion reduction was also observed in the competition experiment. Those results demonstrated that strain LF11 has the capacity of maintaining intestinal barrier and strengthening the defense of pathogens, which was consistent in the traits of probiotics (Yan. et al., 2007). **Figure 2** shows that *B. subtilis* LF11 alone did not cause the survival changes of NCM460 cells dramatically, suggesting that *B. subtilis* LF11 induced neither cytotoxicity nor growth promotion to NCM460 cells. The reductions of the survival of NCM460 cells in exclusion and competition experiments were solely caused by *S. braenderup* H9812 invasion. The higher survival of NCM460 cells in the exclusion experiment than that in competition experiments showed that the pre-exposure was helpful to *B. subtilis* LF11 to play obvious exclusive activity against *Salmonella* infection. Generally, these results are consistent with the reports that probiotics is known to help prevent pathogen infections in application (Que et al., 2021; Yuan et al., 2021).

Probiotics also has the capacity of enhancing the expression and distribution of tight junction proteins to prevent pathogen infection to IECs (Candela et al., 2008; Rao and Samak, 2013; Awad et al., 2017; Blackwood et al., 2017). The tight junction proteins in the intestinal epithelial cells were usually used as biomarkers for the integrity determination of intestinal barrier (Awad et al., 2017; Chelakkot et al., 2018). In this study, the downregulation of both gene transcription and expression levels of four tight junction proteins in NCM460 cells challenged by *S. braenderup* H9812 indicated the damage of the intestinal barrier. In comparison, *B. subtilis* LF11 could upregulate or maintain the gene transcription levels and the expression levels of tight junction proteins in various degrees, indicating that *B. subtilis* LF11 could stimulate the NCM460 cells to strengthen the connection between each cell (**Figures 3A–C**). *B. subtilis* LF11 upregulated the expression of JAM-1 and ZO-1 compared to normal cells, which suggested that *B. subtilis* LF11 enhanced the intestinal epithelium barrier. The various influence on the gene transcription and expression levels of tight junction proteins by *B. subtilis* LF11 may be attributed to their locations and functions in IECs. The CLDNs and OCLN family of transmembrane proteins form the core of the tight junction (Sun et al., 2020). OCLN is localized at the leading edge of cells and regulates directional cell migration and is required for wound healing, whose extracellular domains function in its localization to tight junctions and in regulating the paracellular permeability barrier between cells (Hartsock and Nelson, 2008; Du et al., 2010). CLDNs recruit OCLN to tight junctions, increase epithelial barrier properties, and directly regulate the gate function as paracellular tight junction channels (Hartsock and Nelson, 2008; Markov et al., 2010). In exclusion experiments, *B. subtilis* LF11 prevented the expression downregulation of tight junction proteins and restored the expression levels of CLDN-1 partially and OCLN, JAM-1, and ZO-1 totally, suggesting that the strain LF11 had the capability to enhance the stability and integrity of the intestinal barrier, which agreed with the results described previously (Gong et al., 2016). ZO-1 is a scaffolding protein between transmembrane and cytoplasmic proteins and forms a link between the adherens and tight junctions, which is important for the establishment and maturity of tight junction proteins (Hartsock and Nelson, 2008; Monteiro and Parkos, 2012). The downregulation of gene transcription and expression of ZO-1 protein in NCM460 infected by S. braenderup H9812 alone meant the disruption of tight junction while the upregulation by *B. subtilis* LF11 in the exclusion experiment indicates the enhancement of tight junction. The co-exposure of NCM460 cells to *B. subtilis* LF11 and *S. braenderup* H9812 in competition experiments did not regulate the expression levels of tight junction proteins obviously except for JAM-1. JAM-1 does not directly regulate the barrier but rather functions as a signaling molecule with divergent downstream target proteins to regulate a diverse array of epithelial functions, including epithelial proliferation, migration, and barrier function (Monteiro and Parkos, 2012). Meanwhile, immunofluorescence observation supported the gene transcription and expression analysis (**Figures 4A, B**). The homogeneous distribution and the same levels of the fluorescence intensities of tight junction proteins as the normal IECs proved that *B. subtilis* LF11 possessed protective effects on epithelial barrier integrity against *Salmonella* infection, especially when the *B. subtilis* LF11 was pre-incubated with NCM460 cells.

Cytokines, such as TNF-α and various interleukins, are clearly involved in inflammatory response, inducing redistribution of tight junction proteins (Chiba et al., 2006; Rhayat et al., 2019). Several evidence from mice and animal experiments verified that *B. subtilis* strains had beneficial anti-inflammatory effects (Gong et al., 2016; Rhayat et al., 2019; Liu et al., 2021). Thus, we speculated the effects of *B. subtilis* LF11 on the inflammatory process occurring during *S. braenderup* H9812 infection to IECs. The results in this study confirmed the higher transcription levels of *IL-6*, *IL-8*, and *TNF-α* genes in NCM460 cells infected by *S. braenderup* H9812, which were downregulated by *B. subtilis* LF11 in exclusion and competition experiments. Therefore, the strain LF11 exhibited the regulatory function on epithelial inflammatory factors induced by *S. braenderup* H9812 (**Figures 5A, B**). Meanwhile, the reduction of the IL-8 production supported the transcription analysis. IL-8 could recruit neutrophils into the cecal mucosa to defend against the invasion of *Salmonella in vivo* (Huang, 2021). These findings suggested that probiotic *B. subtilis* LF11 played a role on preventing cytokine-mediated gastrointestinal diseases. Previous studies have shown that proinflammatory cytokines including TNF-α mediate tight junction disruption, which increases the pathogen’s invasion process in epithelial cells (Hatayama et al., 2018). In addition, it was noticed that the exclusion activity was always stronger than competition activity, suggesting that the supplementation with *B. subtilis* LF11 in diets was an effective way to prevent the *Salmonella* infection in poultry farming.

In summary, *B. subtilis* LF11 protected broilers against *Salmonella* infection *via* several ways, including inhibiting the adhesion and invasion of *S. braenderup* H9812 to IECs, enhancing the expression of the tight junction proteins, and attenuating the inflammatory responses of NCM460 cells caused by *S. braenderup* H9812. Those findings indicated that the *B. subtilis* LF11 had the potential as a probiotic for the prevention and treatment of salmonellosis in broilers.

## Data Availability Statement

The original contributions presented in the study are included in the article. Further inquiries can be directed to the corresponding author.

## Author Contributions

RZ and JK contributed conception and design of the study. RZ, ZL, and XG performed the experiments. RZ and XG performed the statistical analysis. RZ, ZL, XG, JZ, TG, and JK wrote and revised the manuscript. All authors contributed to the article and approved the submitted version.

## Funding

This research was funded by the National Natural Science Foundation of China (No. 31871767) and the National Key Research and Development Program of young scientist of China (No. 2019YFA0906700).

## Conflict of Interest

Author RZ was employed by Jinan Scenk Sanfeng Bioengineering Co., Ltd.

The remaining authors declare that the research was conducted in the absence of any commercial or financial relationships that could be construed as a potential conflict of interest.

## Publisher’s Note

All claims expressed in this article are solely those of the authors and do not necessarily represent those of their affiliated organizations, or those of the publisher, the editors and the reviewers. Any product that may be evaluated in this article, or claim that may be made by its manufacturer, is not guaranteed or endorsed by the publisher.
